# Chromosomal Instability in Gastric Cancer: Role in Tumor Development, Progression, and Therapy

**DOI:** 10.3390/ijms242316961

**Published:** 2023-11-30

**Authors:** Marina V. Nemtsova, Ekaterina B. Kuznetsova, Irina V. Bure

**Affiliations:** 1 Laboratory of Medical Genetics, I.M. Sechenov First Moscow State Medical University (Sechenov University), 119991 Moscow, Russia; nemtsova_m_v@mail.ru (M.V.N.); kuznetsova.k@bk.ru (E.B.K.); 2 Laboratory of Epigenetics, Research Centre for Medical Genetics, 115522 Moscow, Russia; 3 Russian Medical Academy of Continuous Professional Education, 125993 Moscow, Russia

**Keywords:** gastric cancer, chromosomal instability, subtype of gastric cancer with chromosomal instability, aneuploidy, structural rearrangements of chromosomal loci, driver genes

## Abstract

According to the Cancer Genome Atlas (TCGA), gastric cancers are classified into four molecular subtypes: Epstein–Barr virus-positive (EBV+), tumors with microsatellite instability (MSI), tumors with chromosomal instability (CIN), and genomically stable (GS) tumors. However, the gastric cancer (GC) with chromosomal instability remains insufficiently described and does not have effective markers for molecular and histological verification and diagnosis. The CIN subtype of GC is characterized by chromosomal instability, which is manifested by an increased frequency of aneuploidies and/or structural chromosomal rearrangements in tumor cells. Structural rearrangements in the CIN subtype of GC are not accidental and are commonly detected in chromosomal loci, being abnormal because of specific structural organization. The causes of CIN are still being discussed; however, according to recent data, aberrations in the *TP53* gene may cause CIN development or worsen its phenotype. Clinically, patients with the CIN subtype of GC demonstrate poor survival, but receive the maximum benefit from adjuvant chemotherapy. In the review, we consider the molecular mechanisms and possible causes of chromosomal instability in GC, the common rearrangements of chromosomal loci and their impact on the development and clinical course of the disease, as well as the driver genes, their functions, and perspectives on their targeting in the CIN subtype of GC.

## 1. Introduction

Gastric cancer (GC) is one of the most common malignant neoplasms, being the fifth-most commonly diagnosed tumor in the world (7% of the total), with the highest incidence in Asia [1]. In most cases, GC is diagnosed at advanced stages; therefore, patients have an unfavorable prognosis and limited treatment options [2].

GC is usually divided into intestinal, diffuse and mixed types according to the Lauren classification, which is based on histopathology. Despite wide clinical application, the Lauren classification does not provide accurate information about the course of tumorigenesis and could not be used for choosing the optimal therapeutic approach [3]. The relative frequencies of intestinal, diffuse and mixed types are approximately 54%, 32% and 15%, respectively [4]. Intestinal and diffuse types of GC are significantly different in etiology, epidemiology, mechanisms of carcinogenesis, biological behavior and prognosis [5]. Diffuse GC is associated with mutations in the *CDH1* gene encoding the cell-adhesion molecule E-cadherin [6] and often demonstrates differential expression of the *RHOA* (Ras homologue family member A) gene, which belongs to the family of small GTPases and participates in cytoskeleton formation and cell adhesion. Intestinal GC is associated with *Helicobacter pylori* infection, which is responsible for the development of more than 60% of GC cases [7,8,9]. The decrease in the infection rate of *H. pylori* in the population of some countries has caused an alteration in the relative frequencies of the GC subtypes, with a significant decrease in intestinal GC and therefore an increase in diffuse GC [10]. Thus, in a population-based study on intestinal and diffuse type GC in the Netherlands between 1989 and 2015, 55% intestinal and 44% diffuse GCs were described [11]. Decreasing incidence of intestinal GC over recent decades has also been reported in Japan [10], the USA [12] and China [13].

The Cancer Genome Atlas (TCGA) research group has proposed the tumor classification system based on profiling of molecular changes in the tumor genome [14]. TCGA has applied approaches based on the analysis of whole-exome sequencing, changes in copy number of specific chromosomal loci, gene expression, DNA methylation, and protein activity in GC [3]. According to the obtained results, GC was classified into four subtypes: Epstein–Barr virus-positive (EBV+) tumors, tumors with microsatellite instability (MSI), tumors with chromosomal instability (CIN) and genomically stable (GS) tumors [14]. The proposed molecular classification facilitates investigations aimed at improving the diagnosis and treatment of GC patients. However, some molecular subtypes of GC, including the CIN subtype, remain insufficiently characterized and do not have valuable and convenient markers for molecular and histological verification [15].

It is known that malignant tumors are characterized by a high level of abnormal genomic changes called genomic instability. Genomic instability includes MSI and CIN [16], and both of these types of instability determine a mutator phenotype of a tumor [17]. MSI is characterized by mutations frequently occurring in areas of microsatellite repeats and is associated with genetic or epigenetic changes in genes encoding the proteins MSH2, MSH6, PMS2 and MLH1, related to the mismatch repair (MMR) system [18]. If genomic changes occur at the chromosomal level, they are called CIN.

CIN can be defined as the existence of spatiotemporal changes in the structure or number of chromosomes that occur during carcinogenesis. These changes are associated with tumor development and can serve as a mechanism for cell adaptation to changes associated with malignant transformation. About 80% of human tumors were found to have chromosomal abnormalities that may indicate the presence of CIN [19]. Aneuploidies and polyploidies often characterize tumors with high metastatic potential, resistance to therapy and poor overall survival, such as triple-negative breast cancer, pancreatic and hepatobiliary cancer, lung cancer, anaplastic thyroid cancer, castration-resistant prostate cancer, low-grade sarcomas, gynecological tumors with serous histology, glioblastoma, cancer of the esophagus and stomach, and microsatellite-stable colorectal cancer [20,21]. Whereas the diagnosis of the GC subtype with MSI is well studied today, the testing for the chromosomal instability subtype of GC is more complex and requires deeper knowledge of the main mechanisms leading to chromosomal instability [22].

In this review, we performed an analysis of the published scientific literature and databases and considered the molecular mechanisms and possible causes of chromosomal instability in GC. The common rearrangements of chromosomal loci and their impact on the development and clinical course of the disease are also addressed, as well as the driver genes, their functions, and perspectives on their targeting in the CIN subtype of GC.

## 2. Types and Mechanisms of Chromosomal Instability in CIN Subtype of GC

CIN is one of the main characteristics of solid tumors. It is considered a variant of genome instability leading to malignant transformation [23]. The CIN subtype of GC is caused by chromosomal instability, which is manifested by an increase in the frequency of aneuploidy and structural chromosomal rearrangements. It is known that chromosomal instability and aneuploidy are not identical, and a cell may be aneuploid, but have a stable karyotype [16,24]. Thus, cells of patients with Patau syndrome (trisomy 13), Edwards syndrome (trisomy 18), Down syndrome (trisomy 21) or Klinefelter syndrome (47, XXY) contain 47 chromosomes and are therefore aneuploid, but do not demonstrate an increased frequency of chromosomal changes. However, the fact that people with Down syndrome have a 200 times higher risk of hematological malignancies confirms associations between aneuploidy and carcinogenesis [25].

**Figure 1:**
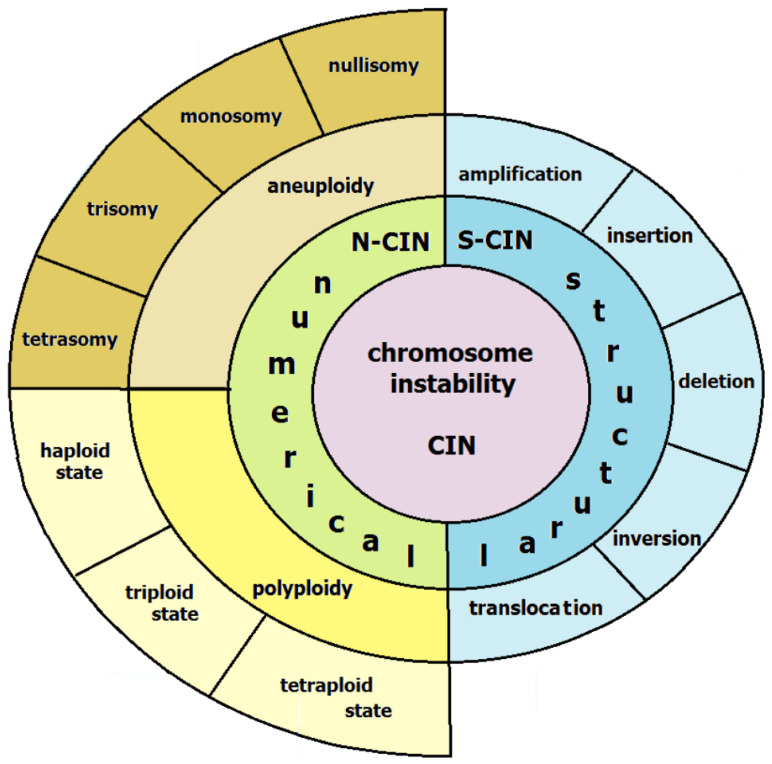
Types of chromosomal instability.

A tumor cell with chromosomal instability is characterized by a high frequency of chromosomal rearrangement accumulation and karyotype instability. CIN can be divided into numerical instability (n-CIN) that is associated with a change in the number of whole chromosomes (aneuploidy) or whole genomes (polyploidy), and structural instability, associated with accumulation of chromosomal rearrangements (s-CIN) (Figure 1).

Numerical changes in chromosomes usually appear as an aneuploidy, a change in the number of chromosomes from nullisomy to tetrasomy, or affect the whole genome, altering its state from haploid to tetraploid. Chromosomal rearrangements in s-CIN are represented by various forms, including deletions, duplications, inversions, insertions, translocations, and by the appearance of dicentric, derivative or ring chromosomes. An increase in the frequency of chromosomal rearrangements leads to deregulations of cell division and chromosome segregation during mitosis, which contributes to an even greater increase in CIN. These changes lead to the loss or increase of genetic material, affecting the expression of genes located in critical areas, and thus have important consequences for tumor growth and progression [26].

The n-CIN and aneuploidy development are caused by mitotic errors that lead to chromosome mis-segregation. Chromosome segregation defects occur as a consequence of mitotic dysfunctions, such as mono- or multipolar spindles, changes in microtubule stability, kinetochore cohesion and assembly defects, or dysfunction of the spindle assembly control point [27]. The main cause of aberrant distribution of whole chromosomes is incorrect attachment, when one kinetochore attaches to microtubules extending from both poles of the spindle [28]. In these cases, there is a lag in the abnormally oriented chromosome in anaphase, which prevents its correct segregation. An important role in the development of CIN is played by aberrations in genes directly involved in regulation of cell division and spindle organization. Among the genes specifically associated with the CIN subtype of GC are *AURKA*, *AURKB*, *CCNB1* and *CDK1*, which are involved in mitosis regulation and cell cycle control. They were described as often amplified or hyperexpressed in CIN subtype [29].

In addition to chromosome mis-segregation, the CIN phenotype can also be caused by deregulation of various cellular processes, including aberrations of cell cycle control points, mitotic stress induced by oncogenes, and DNA replication stress [22].

DNA replication stress is a condition where stopped or slowly progressing replication forks interfere with the timely and correct completion of the S phase, leading to structural instability of chromosomes. Replication stress is caused by both exogenous and endogenous factors, such as DNA damage or the formation of adducts caused by chemical compounds, UV or ionizing radiation, reactive oxygen species, by-products of cellular metabolism, nucleotide pool imbalance, and a lack of replication factors [30]. Another potential cause of replication stress is related to the genetic and epigenetic characteristics of certain chromosomal loci, such as telomeres, centromeres, ribosomal gene loci (rDNA) or fragile sites that are difficult to replicate due to the repetitive sequences that can form secondary structures and alter chromatin conformation [27].

CIN itself does not directly lead to carcinogenesis, but provides more opportunities for avoiding the limitations of proliferative potential and the progressive acquisition of tumor features [31]. Today, views on CIN are changing. It is obvious that CIN promotes carcinogenesis and metastasis; however, CIN also makes the tumor cell more sensitive to certain types of therapy and contributes to immune system activation, directing it to fight tumor cells [32,33].

## 3. Chromosomal Rearrangements Characterizing Chromosomal Instability in Gastric Cancer

Chromosomal rearrangements in the CIN subtype of GC are not accidental and are detected in the chromosomal loci, which are often rearranged because of their structural organization. Such rearrangements are commonly associated with the activation of oncogenes and inactivation of suppressor genes.

**Figure 2:**
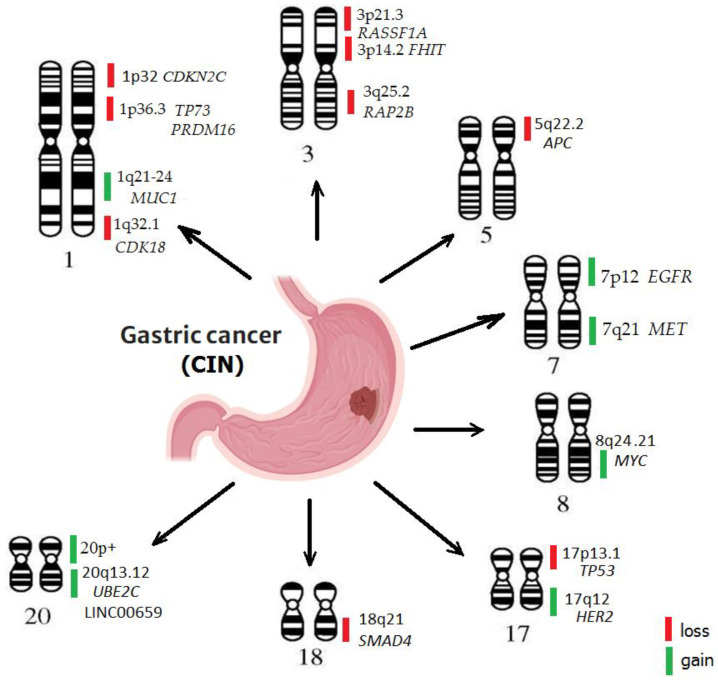
Localization of common chromosomal aberrations in GC.

The description of the GC subtypes has revealed a significantly higher frequency of arm-level copy number variation in the CIN subtype of GC than in the other subtypes (GS, EBV+ and MSI) [14,34]. The greatest differences in frequencies were observed for deletions of loci on chromosomal arms, whereas amplification of chromosomal arms on some chromosomes was observed in all types of GC. The currently available information about specific rearrangements in the CIN subtype of GC is insufficient; however, studies have identified several copy-number variation (CNV) regions that are common for GC. There are frequent gains on chromosomes 1q, 5p, 7, 8, 13 and 20 and losses on chromosomes 1p, 3p, 4, 5q, 9p, 17p, 18q, 19p, 21 and 22 [35] (Figure 2).

Chromosome 1

Loss of genetic material from the short arm of chromosome 1 (1p) is associated with increased tumor aggressiveness and an unfavorable prognosis for patients [36]. The 1p contains several tumor suppressor genes, including *CDKN2C* (1p32.3), which plays an important role in the regulation of the cell cycle. Inactivation or loss of *CDKN2C* can lead to uncontrolled cell proliferation and tumor growth. Locus 1p36.32 contains *TP73*, which is structurally and functionally homologous with *TP53* and is also often lost in GC [37]. Among other genes located in the 1p region and associated with GC is *PRDM16*. It encodes a transcription factor that regulates the state of chromatin and plays an important role in the differentiation of adipose tissue. It is described as an oncoprotein in various types of cancer, including GC [38].

The 1q region is also often rearranged during GC. Homozygous loss at locus 1q32.1 is associated with loss of the *CDK18* gene, a cyclin-dependent protein kinase that phosphorylates proteins involved in the G1–S transition of the mitotic cell cycle. The loss of this protein can lead to deregulation of the cell cycle checkpoint, increased genome instability and development of GC [39]. The *MUC1* gene, a membrane-bound mucin, is also located in the 1q22 region. It plays an important role in protecting gastric epithelial cells from pathogens that initiate inflammation and carcinogenesis. In GC, *MUC1* acts as an oncogene. Being overexpressed in the result of the increased abundance of chromosomal material in the GC tumor tissue, it is associated with aggressive pathological features, deeper invasion, metastasis to the lymph nodes and distant metastases [40].

Chromosome 3

The tumor suppressor genes *FHIT* and *RASSF1A* are located on the short arm of chromosome 3 (3p). The loss of these genes leads to activation of oncogenic pathways, promotes oncogenesis, and is associated with increased metastatic potential and decreased overall survival [41]. The protein encoded by *RAP2B* (3q25.2) is a member of the RAS oncogene family. Its loss contributes to the dysregulation of the RAS pathway, which can cause genomic instability and progression of GC [42].

The *PIK3CA* gene located on chromosome 3q26.3 is often amplified in GC [43], contributing to aberrant proliferation associated with carcinogenesis. The amplification of *PIK3CA* is associated with a poor prognosis, which suggests an important role of this genetic event in the multistage process of gastric carcinogenesis [44].

Chromosome 5

The loss of the long arm of chromosome 5 (5q) is associated with a more invasive phenotype, metastasis to the lymph nodes, and a poor prognosis for GC patients. The 5q contains several tumor suppressor genes, including *APC*. *APC* regulates the Wnt signaling pathway involved in cell proliferation, and its loss leads to activation of the Wnt pathway and promotes tumor growth [45]. Fang et al. have demonstrated that *APC* deletions were detected in 25.9% of GCs and were associated with invasion of lymph nodes or metastases [46].

Chromosome 7

Amplification of the *MET* gene, which is located on 7q21 and encodes the hepatocyte growth factor receptor, was detected in 0–23% of GCs and is associated with late stages of the disease and a poor clinical outcome [47].

An *EGFR* (7p12) copy-number gain is associated with higher risk of invasion and metastasis in solid tumors, including GC [48]. A correlation between the *EGFR* copy-number gain, protein expression and chromosome 7 polysomy was demonstrated [49]. *EGFR* copy-number gain was detected in 22.7% of GC patients and is associated with a poor outcome of the disease [50].

Chromosome 8

A gain in the long arm of chromosome 8q is the most frequent aberration in the CIN subtype of GC. An increase in 8q has been shown to promote cell proliferation, invasion and tumor progression due to the activation of oncogenes located on this chromosome [51]. A common structural change in the CIN subtype of GC is an amplification of the *MYC* gene located on chromosome 8q24. *MYC* encodes a transcription factor regulating cell proliferation, differentiation, and cellular metabolism. Activation and overexpression of *MYC* in GC leads to uncontrolled cell division and tumor growth [52]. MYC amplification was confirmed as an independent prognostic factor for overall survival [53]. The *POU5F1B* gene (POU domain class 5 transcription factor 1B, the OCT4 pseudogene) is located on 8q24 near the *MYC* gene, which is also often amplified in GC. Hayashi et al. have demonstrated that the *POU5F1B* copy number was amplified and overexpressed in GC, and also contributes to an aggressive phenotype and tumor growth [54].

Chromosome 17

Rearrangements of chromosomes 17p and 18q are also significant for the development of the CIN subtype of GC. The *TP53* gene (17p) is a well-known tumor suppressor involved in DNA repair, cell cycle regulation and apoptosis. Mutations or loss of *TP53* allow cells to avoid apoptosis, leading to promotion of tumor growth and development [55]. In an investigation of gastric carcinomas, Zhang et al. identified two states of chromosomal integrity in primary tumors [56]. More than half of the tumors demonstrated aneuploidy and regional CIN, while about a third were chromosomal stable. The authors determined an association of CIN with the loss of function and mutational status of *TP53* and suggested that clinically CIN contributes to increased sensitivity of the tumor to DNA-damaging drugs [56].

Amplification of human epidermal growth factor receptor 2 (*HER2*) is also frequent structural alteration in the CIN subtype of GC. *HER2* is involved in cell growth and proliferation and potentially could be used as a therapeutic target for GC patients [57]. Amplification of *HER2* in GC patients varied from 6% to 23%, and the amplified *HER2* gene was mainly associated with a poor clinical outcome [58]. It has recently been shown that *Helicobacter pylori* CagA induces overexpression of the HER2 protein by *HER2* copy-number gain [59].

Chromosome 18

The loss of 18q is associated with increased tumor aggressiveness and an unfavorable prognosis for the patient [60]. Several tumor suppressor genes—*DCC*, *SMAD4* and *PLCD1*—involved in cell cycle regulation, DNA repair, and angiogenesis are located in this region. Inactivation or loss of these genes leads to deregulation of proliferation, thus contributing to tumor growth [55].

Chromosome 20

Other chromosomal abnormalities that are commonly observed in the CIN subtype of GC include an increase in the copy number in 20q and 20p. Amplified regions on chromosome 20q contain numerous functionally important genes involved in the regulation of the cell cycle (*E2F1*, *TPX2*, *KIF3B*, *PIGT* and *B4GALT5*), DNA methylation and chromatin remodeling (*ASXL1*, *AHCY* and *C20orf20*), and transcription (*TCEA2*) [61]. Amplification of chromosome 20q is associated with deregulation of several specific signaling pathways, including MARK and p53 [62]. In addition, several long noncoding RNAs (lncRNAs) are located in the amplified locus 20q13.33, and are differentially expressed in tumor tissue of GC patients. It has been confirmed that oncogenic lncRNA LINC00659 is activated in GC tissue and associated with the stage of the disease and metastasis to the lymph nodes [63].

The *AURKA* gene (20q13) was found to be often amplified in GC, and its overexpression is associated with worse patient survival [64]. *AUKRA* copy-number gain was detected in 30.5% of GC and was associated with tumor progression, which suggests a prognostic value of *AURKA* [65].

The described chromosomal aberrations in the CIN subtype of GC are summarized in Table 1.

Thus, chromosomal rearrangements are a hallmark of the CIN subtype of GC and can have important consequences for the development of the tumor, its course and response to therapy. The identification of specific genes and genomic loci altered in the CIN subtype of GC is closely related to the development of novel targeted therapies that will lead to better outcomes for patients with this aggressive disease.

## 4. Driver Genes in the CIN Subtypes of GC and Their Associations with Its Tumorigenesis and Therapy

Mutational profiling allows for classification of tumors into specific molecular subtypes according to the spectrum of somatic mutations. Investigation of the somatic mutation profiles in the genes involved in carcinogenesis could help to identify the main or driver mutational events that determine the clinical behavior of the tumor, its aggressiveness, invasion, and metastasis, as well as to find targets for antitumor therapy. Somatic mutations of the tumor genome are usually divided into driver and passenger. Driver mutations provide tumor growth and contribute to the tumor progression. Passenger mutations occur as a consequence of clonal expansion of tumor cells and are not a key event in tumorigenesis. Not only mutations in the driver genes but also their abnormal expression play an important role in tumor development and growth. Driver genes are usually key elements of molecular pathways, and their aberrations are closely related to deregulation of the entire pathological pathway. Today, changes in the GC driver genes in general are rather well described; however, the specific driver mutations for the CIN subtype of GC remain not studied well enough.

### 4.1. Genes Regulating the Cell Cycle and the Response to DNA Damage

According to recent data, *TP53* is not only associated with CIN in GC but also could be the cause of its development [56]. Investigations of the *TP53* mutation profile in GC have confirmed an increase in somatic mutation frequency in chromosomally unstable gastric carcinomas [70]. *TP53* is a tumor suppressor gene that plays a crucial role in the response to DNA damage and cell cycle regulation. Mutations in *TP53* are usually observed in the CIN subtype of GC and are associated with poor prognosis and resistance to chemotherapy and targeted therapy. According to TCGA data, GCs of the CIN subtype usually exhibit an intestinal phenotype and are often *TP53*-mutant (71%), thus proving that the loss of *TP53* function is directly related to the development of the CIN subtype of GC [56]. *TP53* encodes the p53 protein, which is a transcription factor that regulates cell division in response to various stresses, thus acting as a key mechanism of cellular antitumor protection [71]. Most *TP53* mutations are located in the central DNA-binding domain and lead to deregulation of the binding function with the regulatory regions of target genes. Some missense mutations display dominant-negative inhibition of wild-type *TP53*, forming heterodimers with a normal protein, which leads to increased oncogenic function in the absence of wild-type *TP53* [72]. Missense mutations often make the TP53 protein resistant to proteolytic degradation by ubiquitin ligases such as MDM2, COP1, PIRH2 and TRIM24. Their expression is induced by TP53, and therefore they form a negative feedback loop to control stationary protein levels in cells, providing high levels of stable mutant TP53 protein [73].

The *CCNB1* gene encodes the G2/mitotic-specific protein cyclin B1. *CCNB1* has been found to play a significant role in carcinogenesis, especially in GC [74]. Some investigations have shown that *CCNB1* is overexpressed in the CIN subtype of GC. Moreover, expression is observed in both the nucleus and the cytoplasm of the cell [75]. Its overexpression is associated with poor prognosis and reduced overall survival, and targeting *CCNB1* may be a promising therapeutic strategy against GC [76]. *CCNB1* plays a crucial role in the regulation of the cell cycle and oncogenesis in GC, especially in the CIN subtype.

The *CDK1* (cyclin-dependent kinase 1) encodes a key regulator of the cell cycle that plays an important role in the development and progression of various types of cancer, including GC. CDK1 is necessary for transition to the mitotic phase. It is often overexpressed in tumors, and its activity correlates with poor prognosis in cancer patients [77]. CDK1 acts as a kinase that phosphorylates human telomerase reverse transcriptase (hTERT) in T249 during mitosis, which is more common in aggressive and advanced forms of cancer, indicating an additional role of CDK1 in cancer progression. Targeting *CDK1* in combination with conventional therapy could be a promising approach for GC therapy [78]. Inhibition of CDK1 activity can lead to cell cycle arrest and decrease the growth and proliferation of cancer cells. *CDK1* inhibitors cause the cell population to stop in the G1 phase, preventing the transition from G1 to the S phase [79]. *CDK1* inhibition has been shown to selectively inhibit tumor growth in GC cells with *CDKN2A* mutations [80] and in GC mice models with *CDKN2A* mutations [81]. CDK1 inhibitors, such as PD-0332991, have shown promising results in preclinical studies and are undergoing clinical trials for the treatment of advanced GC [82]. Dinaciclib, a complex CDK inhibitor including CDK1, as well as its modern analogues, has demonstrated good antitumor activity in preclinical trials compared with earlier CDK1 inhibitors and suppressed the growth of a wide range of tumors. It was well tolerated in early trials and showed therapeutic effects in patients with solid tumors [83].

### 4.2. Genes Regulating Cell Division

As described above, deregulation of cell division is one of the causes of the CIN subtype of GC development. Therefore, its driver genes include genes involved in the regulation of mitosis. *AURKA* (Aurora kinase A) is located at 20q13 and encodes serine threonine kinase, which phosphorylates an entire network of substrates associated with the regulation of cell division [84]. The AURKA protein plays a role in the formation of microtubules, maintaining the integrity of centrosomes, the spindle of division, and proper cytokinesis [85]; therefore, its amplification and overexpression in the CIN subtype of GC can be considered a cause of chromosomal instability.

Many substrates of AURKA are involved in the organization of mitosis, so the aberrant expression of *AURKA* in various types of tumors is associated with mitotic defects [86]. AURKA phosphorylates Ser10 in the histone H3 tail, initiating mitosis [87]. Phosphorylation of the NDEL1 protein at the Ser251 site by AURKA is necessary for the separation and maturation of centrosomes. Phosphorylated NDEL1 exhibits high affinity for the mitotic protein TACC3, attracting it to the centrosome. The TACC3 protein is also a substrate for AURKA, and it is localized in mitotic spindles after phosphorylation by Ser558 [88]. AURKA activates the NDEL1–TACC3 protein complex and thus plays an important role in the maturation and separation of centrosomes during mitosis. AURKA is also involved in mitosis regulation by interacting with the spindle-associated protein ASAP [89] and centrosome-associated protein CPAP [90]. TPX2 is a substrate for AURKA with phosphorylation sites in Ser121 and Ser125. TPX2 is necessary to establish normal spindle length and interaction with cytoplasmic linker-associated protein 1 [91]. Furthermore, AURKA is responsible for the phosphorylation of Thr210 PLK1, an important mitotic kinase regulating many aspects of the cell division process [92]. Another study showed that CDC25B phosphatase is phosphorylated by AURKA at the Ser353 site, which promotes the G2–M transition [93].

In addition to participating in the mitosis process, AURKA is important for the regulation of β-catenin in GC [94] and also participates in the regulation of TP53 [95]. Phosphorylation of β-catenin by the active kinase GSK-3ß leads to ubiquitination and proteasomal degradation of β-catenin [86].

The *AURKB* gene encodes a mitotic kinase that is involved in cell division and plays an important role in controlling cell proliferation. Recent studies have shown that the functions of AURKB in mitosis include chromosome condensation, formation of a bipolar spindle, and attachment of chromosomes, as well as regulation of the completion of cytoplasmic division [96,97]. Therefore, a violation of the expression of this gene can lead to the development of chromosomal instability in the CIN subtype of GC.

It has been shown that in GC, AURKB participates in the activation of cyclin D1 (CCND1), which regulates the transition from the G1 phase to S phase in the cell cycle. AURKB activates CCND1 expression by phosphorylating histone H3 by Ser10 (H3S10P) on the promoter of the *CCND1* gene [98]. Inhibition of AURKB activity leads to a decrease in H3S10P levels and suppression of CCND1 expression in GC cells, which ultimately leads to inhibition of cell proliferation. Thus, treatment with AZD1152 (barasertib) significantly reduces cell proliferation and formation of cell colonies and leads to G2–M cell cycle arrest in GC cells [96,99].

In addition to the main pathway of CIN development due to the loss of p53 function, amplifications in such signaling pathways as RTK/RAS/MAPK, including HER2, epidermal growth factor (EGFR), MET and FGFR2, were detected in CIN tumors [100].

### 4.3. Genes Regulating EGFR Signaling

*HER2* (human epidermal growth factor receptor 2) is located on chromosome 17 and encodes a transmembrane receptor that plays an important role in GC development and progression. HER2 is a member of the epidermal growth factor (EGFR) receptor family, which stimulates cell growth and division. In normal gastric tissue, HER2 is expressed at a low level, but in some subtypes of GC, including CIN, HER2 can be overexpressed. HER2 overexpression in combination with an amplification of the *HER2* gene is detected in 7–34% of GCs [101]. Its overexpression leads to activation of downstream signaling pathways, such as the phosphatidylinositol-3 kinase (PI3K) pathway and the mitogen-activated protein kinase (MAPK) pathway, which promote the growth and proliferation of tumor cells [102]. Overexpression of HER2 is associated with poor clinical prognosis, including decreased overall survival and increased metastatic potential [103]. However, the increased expression of HER2 is a good therapeutic target for GC treatment with trastuzumab and its analogues. It has been shown that the therapy with trastuzumab deruxtecan (DS-8201) consisting of an antibody against HER2 resulted in a significant improvement in response and overall survival comparing to standard therapy in patients with HER2-positive GC [104].

## 5. The Link between Mutations in Driver Genes and Chromasomal Instability

Currently, the information on driver genes for the CIN subtypes of GC remains insufficient. Well-described driver genes for GC are mostly related to other molecular subtypes. Thus, mutations and aberrations in the genes *CDH1*, *CTNNA1*, and *RHOA*, which regulate cell contact, are often detected in chromosomally stable GC, and mutations in *KRAS*, *PIK3A*, and *ARID1A* are common for the CIN subtype or EB-dependent subtype [14].

Today, CIN is considered a characteristic of not only the CIN subtype of GC but also of various tumor types. Recent studies using genome-wide methods and analysis of a significant number of samples, have attempted to link manifestations of CIN such as change in the copy number and the presence of aneuploidy with patterns of known mutations in driver genes for various types of tumors. It was demonstrated that the genomes of some tumor types differ in a variety of mutational processes, leading to certain patterns of genomic aberrations. The result of such studies led to identification of signatures combining specific chromosomal aberrations and corresponding driver mutations that could correlate with clinical characteristics, such as metastasis, response to treatment, and sensitivity to chemotherapy [105].

In another study, the authors evaluated the degree, diversity and origin of CIN in 7880 tumors representing 33 types of cancer. As a result, 17 signatures were proposed with specific variants of copy-number alterations, which characterized specific types of CIN, their presumed etiology and characteristic damage of pathological pathways, and were associated with the prediction of reactions to certain drugs. In addition, some of these signatures allowed prediction of novel therapeutic targets [106].

**Figure 3:**
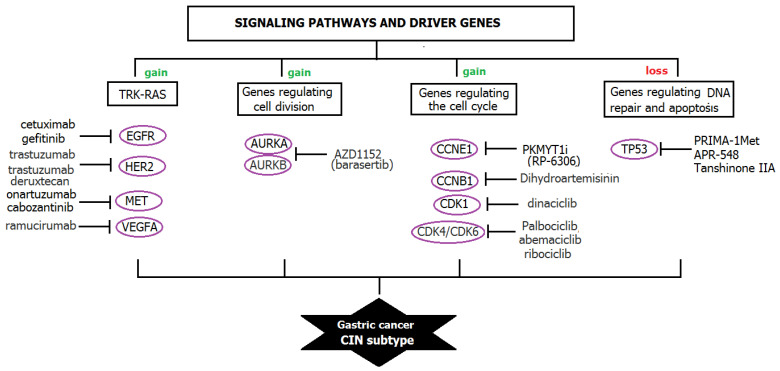
Disruption of signal pathways and frequently mutated driver genes as targets for GC cancer therapy.

Identification of the link between mutations in driver genes and CIN is an important aim directly related to the identification of therapeutic targets. Targeted exposure to frequently mutating driver genes makes it possible to block the active proliferative potential of tumor cells. Many targeted drugs are effectively used to treat various types of tumors, but no sufficiently effective treatment has yet been proposed for GC (Figure 3).

Identification of driver genes is especially important in different patient samples, since national characteristics can alter the lists of identified genes. Comparison of the driver genes obtained by the TCGA studies with the studies of Chinese GC patients has revealed only six genes relevant for both groups [14]. Moreover, in Chinese patients, additional 29 genes were identified that had an increased frequency of mutations in the tumor, and thus could be considered as drivers. Thus, the treatment of patients of different groups may also be slightly different. This should be considered in the development of novel therapeutic agents for targeting driver genes [107].

## 6. Therapeutic Approaches for Treatment of the CIN Subtype of GC

Today, the CIN subtype of GC remains insufficiently studied. The diagnosis of CIN seems to be methodically complex because of the lack of available methods and surrogate markers that would allow determining damages in chromosomes and genomes.

Therapy of the CIN subtype of GC involves multimodal and personalized approaches based on the individual molecular characteristics of the patient’s tumor. Advances in targeted therapy allow the use of novel drugs that can improve the overall survival and quality of life of patients with the CIN subtype of GC. For effective targeted therapy of the CIN subtype of GC, knowledge of alterations in driver genes should be used, as well as the most common molecular pathways that have been identified as potential therapeutic targets. One such direction is the targeting of the TP53 pathway, which is often mutated in the CIN subtype of GC. Today, several drugs are being tested that restore the function of the *TP53* gene in tumor cells (APR-548), as well as drugs that can reactivate the function of mutated *TP53* and induce apoptosis of tumor cells (PRIMA-1Met) [108,109]. Another direction is targeting the pathways of receptor tyrosine kinases (RTKs), which are hyperactivated in the CIN subtype of GC. Trastuzumab and cabozantinib, which target *HER2* and *MET*, respectively, are currently approved as potential treatment options for GC [110].

However, the clinical characteristics of the CIN subtype are already known, allowing prediction of the course of the tumorigenesis and offering certain options for therapy. Such characteristics include a more aggressive course of the tumor with CIN. According to available data, the CIN subtype is characterized by a poor prognosis and reduced patient survival [111,112]. At the same time, more than 40% of GCs have a high level of PD-L1 expression [113]. Immune checkpoint inhibitors such as avelumab, nivolumab, and pembrolizumab are currently approved for the treatment of various malignant neoplasms and have demonstrated promising results in therapy of the CIN subtype of GC [114].

The CIN subtype of GC is also characterized by low infiltration of the tumor by immune cells. It has been shown that immunosuppression is observed in patients with the CIN subtype, which may be one of the main mechanisms explaining poor survival and may become the target for novel therapeutic solutions [115]. An additional feature of the CIN subtype is the state of increased intratumor heterogeneity, which can affect the clinical course of the disease and is associated with the effectiveness of therapeutic approaches. Intratumor heterogeneity complicates application of targeted drugs, leaving intact insensitive cells that can form a relapse. It is known that CIN leads to multidrug resistance [116], and single-drug strategies usually fail due to the ineffectiveness of treatment of the entire tumor population and the adaptive nature of cells exhibiting CIN. However, on the other hand, an increase in the frequency of chromosomal aberrations increases the sensitivity of cells to cytotoxic drugs and radiotherapy. It was hypothesized that there is an optimal level of CIN that supports tumor growth, but exceeding this level and the accumulation of significant chromosomal changes leads to a significant decrease in the viability of tumor cells and an improved response to cytotoxic treatment [117]. Today, due to the progress in molecular technologies that allow the determination of CIN in tumor cells, interest in the CIN subtype of GC has grown significantly.

One of the promising technologies in field of molecular oncology today is high-throughput parallel sequencing. Somatic mutation profiling of tumor tissue is applied in practical oncology to study mutations in driver genes, search for drug targets, and determine mutational load and microsatellite instability [107]. The search for a mutational or expression profile in tumor tissue associated with chromosomal instability could allow for new variants of biomarkers associated with the CIN subtype of GC to be proposed.

Complex genomic profiling by using next-generation sequencing of tissue or circulating tumor DNA is already being applied in some countries to study biomarkers associated with the subsequent treatment of GC [118]. Presumably, a comprehensive molecular profiling strategy will contribute to the wider use of precision medicine and molecular-guided therapy in patients with GC.

## 7. Conclusions

Since CIN is an important mechanism by which tumor cells exhibit genomic instability, efforts aimed at identifying and characterizing the main stages and mechanisms of CIN in GC will improve understanding of the biology of tumor transformation. CIN investigation will be important for improving the risk stratification of GC patients, increasing the therapeutic response and clinical outcomes. However, a significant obstacle in this endeavor is our limited capabilities and technical problems associated with measuring CIN in a clinical setting. Technological progress and its clinical use will make it possible to gain an understanding of the molecular mechanisms of CIN and its role in carcinogenesis, and to develop new therapeutic strategies aimed at improving the life and prognosis of GC patients.

## Figures and Tables

**Table 1:** Chromosomal aberrations in GC.

Gene	Location	Function	Role in Pathogenesis	Refs.
*TP73*	1p36.32	cell cycle regulation, induction of apoptosis	mutations are associated with GC progression	[37]
*PRDM16*	1p36.3	regulation of transcription	high copy gain is associated with late cancer stages and poor prognosis	[38]
*CDK18*	1q32.1	regulation of cell cycle and proliferation	gene inactivation promotes GC progression	[39]
*MUC1*	1q21-24	regulation of apoptosis, protection of gastric epithelial cells from pathogens that initiate inflammation and carcinogenesis	high copy gain is associated with deeper invasion and metastasis	[40]
*RASSF1A*	3p21.3	regulation of cell cycle; inhibits epithelial-mesenchymal transition	gene inactivation is associated with metastasis and poor overall survival	[41]
*FHIT*	3p14.2	regulation of apoptosis	associated with tumor progression and poor prognosis	[66]
*RAP2B*	3q25.2	regulation of proliferation, migration, invasion and apoptosis after DNA damage	gene inactivation promotes GC progression	[38]
*PIK3CA*	3q26.3	regulation of proliferation	gene amplification is associated with poor prognosis	[43,44]
*APC*	5q22.2	regulation of the mitotic spindle to facilitate proper chromosome segregation	deletion is associated with tumor progression and poor prognosis	[42]
*EGFR*	7p12	regulation of cellular growth, proliferation, and differentiation	copy number gain is associated with higher risk of invasion and metastasis	[48,50]
*MET*	7q21	regulation of cell motility	gene amplification is associated with late stages and poor prognosis	[47]
*MYC*	8q24.21	regulation of cell proliferation and differentiation	promotes invasion and tumor progression	[52]
*POU5F1B*	8q24	regulation of angiogenesis, cell proliferation and apoptosis	copy number gain is associated with aggressive phenotype	[54]
*TP53*	17p13.1	regulation of cell cycle, DNA repair and apoptosis	mutations in gene are associated with longer overall survival	[55]
*HER2*	17q12	regulation of cell growth and proliferation	amplification of gene promotes GC	[57]
*UBE2C*	20q13.12	regulation of proliferation and cell cycle	associated with poor outcome	[67,68]
*AURKA*	20q13	regulation of DNA repair, cell migration and invasion	amplification of gene is associated with poor patient survival	[64,65]
*C20orf20*	20q13.33	regulation of DNA methylation and chromatin remodeling	associated with high tumor aggressiveness	[61,69]
LINC00659	20q13.33	regulation of migration and invasion	associated with the stage of the disease and metastasis to the lymph nodes	[63]
